# Breastfeeding practices among patients managed by a comprehensive cardio-obstetrics program

**DOI:** 10.1080/14767058.2023.2253485

**Published:** 2023-12

**Authors:** Isabel C. Collins, Christina T. Blanchard, Ayamo Oben, Ashton Robinson, Tavonna Kako, Joanna M. Joly, Marc G. Cribbs, Brian Casey, Alan Tita, Rachel Sinkey

**Affiliations:** a Department of Obstetrics and Gynecology, University of Alabama at Birmingham, Heersink School of Medicine, Birmingham, AL;; b Department of Obstetrics & Gynecology, University of Alabama at Birmingham, Birmingham, AL;; c Center for Women’s Reproductive Health, University of Alabama at Birmingham, Birmingham, AL;; d Department of Medicine, Division of Cardiovascular Disease, University of Alabama at Birmingham, AL

**Keywords:** Breastfeeding, maternal cardiac disease, postpartum visit

## Abstract

**Objective:**

To evaluate breastfeeding intent, rates at discharge, and continued breastfeeding at follow-up in patients managed in a comprehensive cardio-obstetrics program stratified by severity of maternal cardiac disease.

**Study Design:**

Retrospective cohort of patients managed by a comprehensive cardio-obstetrics program at the University of Alabama at Birmingham (UAB). Patients were included if they had ≥1 prenatal visit with the Cardio-Obstetrics team and delivered at UAB. The primary outcome was the breastfeeding rate on discharge from the delivery-associated hospitalization. Secondary outcomes included intent to breastfeed on admission and breastfeeding at the postpartum visit. Baseline characteristics and rates were compared between patients with less severe (mWHO I – II/III) vs. more severe (mWHO III – IV) maternal cardiac disease.

**Results:**

147 patients were included: 85 (57.8%) mWHO class I – II and 62 (42.2%) mWHO class III–IV. Patients with more severe maternal cardiac disease had higher rates of chronic hypertension (22.6% vs. 9.4%; *p* = 0.027), lower gestational age at delivery (36.4 vs 37.7 weeks; *p* = 0.008), and higher rates of NICU admission (31.2% vs. 14.1%; *p* = 0.013). There were no significant differences between mWHO class I-II vs. mWHO class III-IV in intent to breastfeed upon admission to the delivery-associated hospitalization (84.7% vs. 82.3%; *p* = 0.67), breastfeeding rates upon discharge from the delivery-associated hospitalization (90.6% vs. 87.1%; *p* = 0.50), or breastfeeding rates at the postpartum visit (54.1% vs. 48.5%; *p* = 0.60).

**Conclusions:**

Despite potential barriers in this high-risk population, over 85% of patients breastfed upon discharge from the delivery-associated hospitalization. However, breastfeeding rates dropped by 40% at the postpartum visit. Strategies to support breastfeeding in the post-partum period in patients with maternal cardiac disease are imperative.

## Introduction

Breastfeeding confers many physiologic benefits for both the mother and neonate [1]. One systematic review demonstrated a reduction in breast cancer risk and improved birth spacing with a possible reduction in ovarian cancer and diabetes [2]. Studies have demonstrated the reduction of cardiovascular risk factors such as obesity, hypertension, hyperlipidemia, and diabetes in the postpartum period in breastfeeding women [3,4]. This reduction in cardiovascular risk factors may be due to the increased metabolic requirements of breastfeeding, improved pancreatic beta cell function, increased levels of HDL, and lower levels of triglycerides and cholesterol [1]. However, there is a gap in knowledge regarding breastfeeding practices in women with heart disease. A recent Canadian study demonstrated twice the odds of early cessation of exclusive breastfeeding in women with chronic diseases including arthritis, scoliosis, fibromyalgia, osteoporosis, high blood pressure, heart diseases, and diabetes [5]. Another study investigating postpartum outcomes in patients with congenital heart disease reported lower rates of breastfeeding in those with more severe cardiac disease [6]. Additional data regarding breastfeeding intent, rates, or duration specific to women with maternal cardiac disease are needed to better inform interventions in this population.

Therefore, our objective is to evaluate breastfeeding practices defined by breastfeeding intent, prevalence of breastfeeding at discharge from delivery-associated hospitalization, and continued breastfeeding at follow-up in patients managed in a comprehensive cardio-obstetrics program stratified by severity of maternal cardiac disease. We hypothesized that despite no difference in breastfeeding intent, women with severe heart disease (defined as mWHO Class III/IV) would be less likely to breastfeed than women with less severe heart disease (defined as mWHO Class I - II/III) both at discharge from the delivery-associated hospitalization as well as at the postpartum visit.

## Methods

We performed a single-center retrospective cohort study of women with cardiac disease managed by the University of Alabama at Birmingham (UAB) Cardio-Obstetrics Program. The program is a collaboration between the Division of Maternal-Fetal Medicine and the Division of Cardiovascular Medicine at UAB to provide comprehensive, multidisciplinary prenatal care to pregnant individuals with congenital and acquired heart disease. Approval for the study was obtained through the UAB Institutional Review Board (IRB-300002012). Informed consent was waived given the retrospective nature of the study. Patients were identified from the cardio-obstetrics database and included if they had ≥1 prenatal visit with the program and delivered at UAB between 03/01/2015 and 6/30/19; intrauterine fetal demise or death of the neonate prior to postpartum visit were considered exclusion criteria.

Baseline demographics were collected from the medical record (Supplemental Table 1). Patients were stratified by maternal cardiac disease (MCD) severity defined by a modified World Health Organization (mWHO) classification [7,8]. The mWHO classification is endorsed by the American Heart Association and the American College of Obstetricians and Gynecologists (ACOG). Briefly, less severe lesions are graded as class I, conditions that pose a threat to the life of the mother are class IV, and classes in between are of moderate risk. The mWHO classification was defined per ACOG and independently confirmed by an adult congenital cardiologist (MGC).

The primary outcome was breastfeeding, defined as an expression of maternal human milk and administered to the neonate *via* either direct feeding or pumping, at discharge from the delivery-associated hospitalization. Secondary outcomes included self-reported intent to breastfeed at admission for the delivery-associated hospitalization and self-reported breastfeeding at the postpartum visit between 4–8 weeks postpartum. Outcomes were abstracted by our team (ICG, AR) from the admission History and Physical, hospital progress notes, and postpartum clinic notes.

Differences between groups were compared using Student *t* tests and Wilcoxon rank sum tests for continuous variables, while categorical variables were assessed, as applicable, using χ^2^ and Fisher’s exact tests. All analyses were performed using SAS version 9.4 (Cary, NC) and assessed at a significance level of *p* < 0.05.

## Results

One hundred forty-seven patients managed by the Cardio-Obstetric Program at UAB were included: 85 (57.8%) mWHO class I-II/III and 62 (42.2%) mWHO class III-IV. While 156 patients were identified in the database, *n* = 9 were excluded due to lack of mWHO classification. Patients with mWHO class III – IV heart disease had higher rates of chronic hypertension (22.6% vs. 9.4%; *p* = 0.03), lower gestational age at delivery (36.4 vs 37.7 weeks; *p* = 0.01), and higher rates of NICU admission (31.2% vs. 14.1%; *p* = 0.01). They were also less likely to be married/living with a partner (51.2% vs 32.3%; *p* = 0.02). Remaining baseline characteristics were not different between groups (Supplemental Table 1).

**Figure 1.**
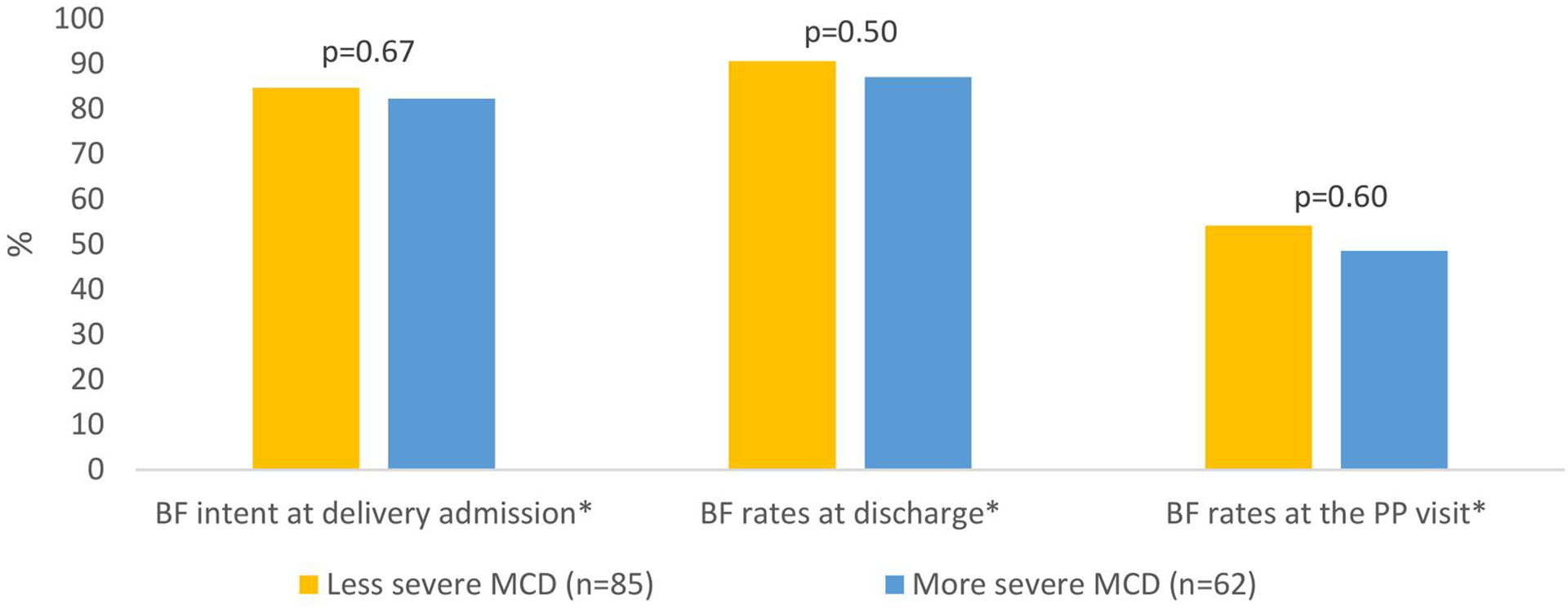
Breastfeeding practices in patients managed by the UAB Cardio-Obstetrics Program.

There were no significant differences between groups with less severe (mWHO class I-II/III) and more severe (mWHO class III/IV) disease, respectively, in intent to breastfeed (84.7% vs. 82.3%; *p* = 0.67), breastfeeding rates at time of discharge from delivery-associated hospitalization (90.6% vs. 87.1%; *p* = 0.50), or breastfeeding rates at the post-partum visit (54.1% vs. 48.5%; *p* = 0.60). Attendance of the postpartum visit in patients with less severe cardiac disease was 76.5% while in the more severe cardiac disease group it was 64.5% (*p* = 0.11). In both groups, there was a decrease in breastfeeding between hospital discharge and the postpartum visit. Breastfeeding rates decreased by 36.5% and 38.6% in patients with less and more severe cardiac disease, respectively (Figure 1, Supplemental Table 2).

## Discussion

Despite potential breastfeeding barriers among patients with maternal cardiac disease, the severity of cardiac disease had no significant impact on breastfeeding practices in patients managed by UAB’s Cardio-Obstetrics Program. These results differ from the aforementioned studies, which demonstrate differences in breastfeeding practices based on the presence and/or severity of chronic disease [5,6]. Rates of breastfeeding at about 82–85% in our population are comparable to the 83% rate in the United States and higher than the 71% rate reported in the state of Alabama by the CDC in the 2022 Breastfeeding Report Card [9]. We suspect that this is due to increased support and successful interventions in the inpatient setting, specifically *via* the widespread utilization of lactation consultants. We acknowledge that the sample size could be underpowered to detect a difference and that these data do not provide granul-specific cardiac-specific lesions, for example, decompensated heart failure.

Importantly, there was a significant decrease in breastfeeding rates at the post-partum visit in our population. These results, while limited by potential selection bias due to loss of follow-up, signify the need for better interventions to support mothers who desire to breastfeed immediately after discharge. The literature has demonstrated an improved cardiovascular risk profile associated with women who breastfeed [3,4]. Given the available literature detailing a higher prevalence of adverse postpartum cardiovascular outcomes in women with heart disease [10], it is important to address the potential benefits and challenges of breastfeeding practice in the postpartum period in patients with maternal cardiac disease. Potential strategies that may enhance breastfeeding rates in the postpartum period include longer maternity leave, funding for mothers during the postpartum period, workplace accommodations when they return to work, and wider access to pumping equipment. Additionally, we have an ongoing project funded by the American Heart Association Health Equity Research Network to test whether digital health and/or community health worker interventions can improve outcomes including breastfeeding rates. While this study is not focused solely on mothers with cardiac disease, we hope that these interventions can be applied in the future to mothers with heart disease.

## Conclusion

This study represents a well-characterized cohort of patients and data derived from the UAB Cardio-Obstetrics Program and provides insight into an increasingly prevalent subset of cardio-obstetrics patients in which breastfeeding rates have not been well-characterized. While the severity of heart disease did not significantly affect breastfeeding practices in our population of women with maternal cardiac disease, a decrease in rates of breastfeeding at the post-partum visit identifies important opportunities for future research and interventions. Strategies to support breastfeeding following hospital discharge in this population are imperative.

## Supplementary Material

Supplementary Material

## Figures and Tables

**Table 1.** Baseline characteristics of patients managed by a comprehensive Cardio-Obstetrics Program.

CHARACTERISTIC	Less severe (mWHO Class I–II) (*n* = 85)	More severe (mWHO Class III–IV) (*n* = 62)	*p*-value
Maternal age	27.4±6.7	27.2±6.3	0.91
Race/ethnicity^a^			0.56
Black, non-Hispanic	33 (39.3)	25 (40.3)	
White, non-Hispanic	48 (57.1)	32 (51.6)	
Hispanic	3 (3.6)	4 (6.5)	
Asian	0 (0.0)	1 (1.6)	
Parity			0.36
Nulliparous	36 (42.4)	31 (50.0)	
Multiparous	49 (57.7)	31 (50.0)	
BMI	31.4±8.3	33.1±8.7	0.24
Married or living with partner^a^	**43 (51.2)**	**20 (32.3)**	**0.02**
Private Insurance	37 (43.5)	20 (32.3)	0.15
GA at delivery^a^	**37.7±2.7**	**36.4±3.5**	**0.01**
Cesarean Delivery	40 (47.1)	25 (40.3)	0.42
NICU Admission^a^	**12 (14.1)**	**19 (31.2)**	**0.01**
GHTN/Preeclampsia	21 (24.7)	17 (27.4)	0.71
CHTN	**8 (9.4)**	**14 (22.6)**	**0.03**
DM	5 (5.9)	7 (11.3)	0.24

BMI: body mass index; CHTN: chronic hypertension; DM: diabetes mellitus; GA: gestational age; GHTN: gestational hypertension, mWHO: modified world health organization; NICU: neonatal intensive care unit.

a Variables missing data include race *n* = 1, married or living with partner *n* = 1, NICU *n* = 1.

**Table 2.** Breastfeeding practices in patients managed by a comprehensive Cardio-Obstetrics Program.

OUTCOMES	Less severe (mWHO class I-II) (*n* = 85)	More severe (mWHO class III-IV) (*n* = 62)	*p*-value
Intent to breastfeed^a^ at admission for delivery			0.67
Formula feeding	8 (9.4)	5 (8.1)	
Any Breastfeeding (Breast or Both)	72 (84.7)	51 (82.3)	
Unknown/Undecided	5 (5.9)	6 (9.7)	
Breastfeeding^a^ rates at discharge from the delivery-associated hospitalization	77 (90.6)	54 (87.1)	0.50
Breastfeeding^a^ rates at the postpartum visit	33 (54.1)^b^	16 (48.5)^b^	0.60
Attended postpartum visit	65 (76.5)	40 (64.5)	0.11

mWHO: modified world health organization.

a Breastfeeding is defined as expression of maternal human milk *via* direct feeding or pumping.

b BF rates at the postpartum visit were calculated as a percentage with the denominator being women who attended the postpartum visit and had breastfeeding status documentation.

## Data Availability

The authors confirm that the data supporting the findings of this study are available within the article [and/or] its supplementary materials.
