# Supplementary material for: Breastfeeding practices among patients managed by a comprehensive cardio-obstetrics program
Source: J Matern Fetal Neonatal Med. Author manuscript; Available in PMC 2023 Dec 1. (PMC10580703; doi:10.1080/14767058.2023.2253485)
Supplement: Supplementary Material [file NIHMS1936809-supplement-Supplementary_Material.pdf]

Supplemental Materials:

Table 1. Baseline characteristics of patients managed by a comprehensive Cardio-Obstetrics Program.

CHARACTERISTIC	Less severe (mWHO Class I-II) (n = 85)	More severe (mWHO Class III-IV) (n = 62)	p-value
Maternal age	27.4 ± 6.7	27.2 ± 6.3	0.91
Race/ethnicity*			0.56
Black, non-Hispanic	33 (39.3)	25 (40.3)	
White, non-Hispanic	48 (57.1)	32 (51.6)	
Hispanic	3 (3.6)	4 (6.5)	
Asian	0 (0.0)	1 (1.6)	
Parity			0.36
Nulliparous	36 (42.4)	31 (50.0)	
Multiparous	49 (57.7)	31 (50.0)	
BMI	31.4 ± 8.3	33.1 ± 8.7	0.24
Married or living with partner*	**43 (51.2)**	**20 (32.3)**	**0.02**
Private Insurance	37 (43.5)	20 (32.3)	0.15
GA at delivery*	**37.7 ± 2.7**	**36.4 ± 3.5**	**0.01**
Cesarean Delivery	40 (47.1)	25 (40.3)	0.42
NICU Admission*	**12 (14.1)**	**19 (31.2)**	**0.01**
GHTN/Preeclampsia	21 (24.7)	17 (27.4)	0.71
CHTN	**8 (9.4)**	**14 (22.6)**	**0.03**
DM	5 (5.9)	7 (11.3)	0.24

BMI = body mass index, CHTN = chronic hypertension, DM = diabetes mellitus, GA = gestational age, GHTN = gestational hypertension, mWHO = Modified World Health Organization, NICU = neonatal intensive care unit

* Variables missing data include race n=1, married or living with partner n=1, NICU n=1

Table 2. Breastfeeding practices in patients managed by a comprehensive Cardio-Obstetrics Program

OUTCOMES	Less severe (mWHO class I-II) (n=85)	More severe (mWHO class III-IV) (n=62)	p-value
Intent to breastfeed at admission for delivery			0.67
Formula feeding	8 (9.4)	5 (8.1)	
Any Breastfeeding (Breast or Both)	72 (84.7)	51 (82.3)	
Unknown/Undecided	5 (5.9)	6 (9.7)	
Breastfeeding rates at discharge from the delivery-associated hospitalization	77 (90.6)	54 (87.1)	0.50
Breastfeeding rates at the postpartum visit	33 (54.1)**	16 (48.5)**	0.60
Attended postpartum visit	65 (76.5)	40 (64.5)	0.11

mWHO = Modified World Health Organization

*Breastfeeding is defined as expression of maternal human milk via direct feeding or pumping

**BF rates at the postpartum visit were calculated as a percentage with the denominator being women who attended the postpartum visit and had breastfeeding status documentation
